# Biophysical, Biochemical, and Photochemical Analyses Using Reflectance Hyperspectroscopy and Chlorophyll a Fluorescence Kinetics in Variegated Leaves

**DOI:** 10.3390/biology12050704

**Published:** 2023-05-11

**Authors:** Renan Falcioni, Werner Camargos Antunes, José A. M. Demattê, Marcos Rafael Nanni

**Affiliations:** 1 Department of Agronomy, State University of Maringá, Av. Colombo, 5790, Maringá 87020-900, Paraná, Brazil; wcantunes@uem.br (W.C.A.); mrnanni@uem.br (M.R.N.); 2 Department of Soil Science, Luiz de Queiroz College of Agriculture, University of São Paulo, Av. Pádua Dias, 11, Piracicaba 13418-260, São Paulo, Brazil; jamdemat@usp.br

**Keywords:** *Codiaeum variegatum*, JIP test, modeling pigment, nonphotochemical quenching, optical spectroscopy, phenomenological modeling, photosynthesis, variegate leaves, vegetation indexes

## Abstract

**Simple Summary:**

This study investigates the spatial analysis of morphological and chemical changes and how reflectance hyperspectroscopy and fluorescence kinetics spectroscopy can enhance our understanding of biophysical, biochemical, and photochemical changes in *Codiaeum variegatum* (L.) A. Juss, a plant with variegated leaves and different pigments. The analysis included pigment profiling, hyperspectral curves, chlorophyll a fluorescence induction kinetics, and multivariate analyses associated with 23 JIP test parameters and 34 vegetation indexes. The results show that the analysis of chemical composition combined with vegetation indexes, such as PRI, PSSRc, ARI1, RARS, and SIPI, are highly correlated with pigment concentration and photochemical components of photosystems in leaves. Furthermore, decreased damage to energy transfer in the electron transport chain is associated with the accumulation of carotenoids, anthocyanins, flavonoids, and phenolic compounds linked with specific wavelengths. Our results reveal the potential of optical spectroscopy techniques and multivariate data analyses to enhance the management and monitoring of the leaf color status of plants. This is the first study and report on the monitoring of nonuniform leaves, particularly in the alteration of photosystem changes in variegated leaves together with high-throughput analyses.

**Abstract:**

The adjustments that occur during photosynthesis are correlated with morphological, biochemical, and photochemical changes during leaf development. Therefore, monitoring leaves, especially when pigment accumulation occurs, is crucial for monitoring organelles, cells, tissue, and whole-plant levels. However, accurately measuring these changes can be challenging. Thus, this study tests three hypotheses, whereby reflectance hyperspectroscopy and chlorophyll a fluorescence kinetics analyses can improve our understanding of the photosynthetic process in *Codiaeum variegatum* (L.) A. Juss, a plant with variegated leaves and different pigments. The analyses include morphological and pigment profiling, hyperspectral data, chlorophyll a fluorescence curves, and multivariate analyses using 23 JIP test parameters and 34 different vegetation indexes. The results show that photochemical reflectance index (PRI) is a useful vegetation index (VI) for monitoring biochemical and photochemical changes in leaves, as it strongly correlates with chlorophyll and nonphotochemical dissipation (Kn) parameters in chloroplasts. In addition, some vegetation indexes, such as the pigment-specific simple ratio (PSSRc), anthocyanin reflectance index (ARI1), ratio analysis of reflectance spectra (RARS), and structurally insensitive pigment index (SIPI), are highly correlated with morphological parameters and pigment levels, while PRI, moisture stress index (MSI), normalized difference photosynthetic (PVR), fluorescence ratio (FR), and normalized difference vegetation index (NDVI) are associated with photochemical components of photosynthesis. Combined with the JIP test analysis, our results showed that decreased damage to energy transfer in the electron transport chain is correlated with the accumulation of carotenoids, anthocyanins, flavonoids, and phenolic compounds in the leaves. Phenomenological energy flux modelling shows the highest changes in the photosynthetic apparatus based on PRI and SIPI when analyzed with Pearson’s correlation, the hyperspectral vegetation index (HVI) algorithm, and the partial least squares (PLS) to select the most responsive wavelengths. These findings are significant for monitoring nonuniform leaves, particularly when leaves display high variation in pigment profiling in variegated and colorful leaves. This is the first study on the rapid and precise detection of morphological, biochemical, and photochemical changes combined with vegetation indexes for different optical spectroscopy techniques.

## 1. Introduction

Plants are constantly exposed to varying light conditions, which can lead to limitations in biophysical, biochemical and photochemical processes of photosynthesis. This results in natural physiological adjustments in both the photosystems and the plant as a whole during leaf accumulation of pigments and the advancement of variegated leaf development [1,2]. These changes in photosystems can be measured directly or indirectly from chloroplasts to leaves to whole-plant levels, helping to identify injuries and stresses, understand spectral modifications in leaf optical properties, and comprehend pigment biosynthesis and degradation, as well as changes in the electron transport chain and membrane energy flow [3,4,5,6]. Current research focuses on structural, biochemical, and spectral changes in different anatomical and morphological leaves [2,7], which are important for understanding biological communities, agricultural productivity, and monitoring ecosystems.

The Euphorbiaceae family is abundant and encompasses 6000 species and 300 different plant types. The *Codiaeum* genus, part of this family, includes 46 species found in tropical and subtropical regions worldwide. *Codiaeum variegatum* (L.) A. Juss is known for its naturally variegated leaves resulting from a combination of genetic and environmental factors. In this way, variegation in leaves is characterized by color variations, particularly in normally green tissues, leading to changes in photosynthetic parameters, chlorophyll fluorescence, and reflectance factors [8,9]. *C. variegatum* (L.) A. Juss has normal chloroplasts in its green sections, but exhibits significant changes in chloroplasts and thylakoid membranes in its yellow sections [10]. Furthermore, orange and red–purple sections have chlorophylls in chloroplasts influenced by interactions among phenolic compounds, anthocyanins and flavonoids [5,11].

Leaves are essential for photosynthesis and directly interact with incoming light. They contain molecules that promote energy absorption and reflection, and can modify the leaf’s optical properties and alter the pigment content and concentration [10]. In this way, different bands of the electromagnetic spectrum have distinct biochemical, biophysical, or structural characteristics that provide insights into physiology through spectroscopy analyses [5,6]. On the other hand, pigments absorb radiation in the ultraviolet-visible spectrum (350–700 nm), while the NIR (700–1300 nm) and SWIR (1300–2500 nm) bands are influenced by cellular components such as cellulose, lignin, nitrogen concentrations, and leaf water levels [12,13]. Therefore, VIS–NIR–SWIR analysis has greatly contributed to identifying plant communities and unique species by monitoring biochemical and photochemical changes from chloroplasts to leaves [5,6,14,15].

Hyperspectroscopy techniques, including vegetation index (VI) analyses, are widely used in ecophysiology, environmental research, agricultural monitoring, plant distribution, and ecosystem studies. These techniques provide insights into biochemical and physiological processes in plants and vegetation, as well as ecological interactions on local and global scales [14,16,17,18]. For example, VIs offer valuable information about the chemical and physiological-photosynthetic parameters of plants, including dark respiration, dissipation by photochemical and nonphotochemical processes, stomatal conductance, and water use efficiency [14,19,20,21]. In addition, few studies have demonstrated the usefulness of various vegetation indexes as predictors of optical and spectral properties, as well as the status of plant growth over time [22,23,24,25].

Chlorophyll fluorescence (ChF) techniques, including the JIP test, should assess the photosynthetic profile and electron transport chain points. The JIP test provides a comprehensive view of photosynthetic apparatus conditions using high-resolution measurements of fast fluorescence [9] to calculate various parameters on a one-second time scale [26,27]. These parameters describe energy flows in and around photosystem II (PSII) reaction centers [9,28,29], including active cross sections (CSs) and phenomenological energy flows. The JIP test parameters evaluate consecutive photon absorption energy flows (ABS), excitation capture (TR), energy dissipation (DI), electron transport (ET), and the reduction of final electron acceptors on the PSI acceptor. Additionally, PSII behavior parameters define quantum yields and efficiencies, such as the maximum quantum yield of primary photochemistry (φPo), quantum yield of electron transport (ΦEo), trapped exciton probability of electron transfer to ET/CS beyond QA (ΨEo), and electron transfer probability to reduce the final electron acceptors on the PSI acceptor side (δRo) [9,28,29,30]. However, it is unclear whether the JIP test correlates with VIs and how hyperspectral tools can determine this status.

This study aims to determine the effectiveness of using vegetation indexes (VIs) as an alternative to monitoring morphological, biochemical and photochemical information in variegated leaves. For this purpose, high-resolution equipment, including hyperspectral data and fluorescence techniques via the JIP test, was used to test three hypotheses. First, the study hypothesizes that hyperspectral reflectance curves can evaluate, separate, and correlate changes in morphological, pigment content, and photochemical activity in colorful leaves with VIs. Second, chlorophyll a fluorescence kinetics curves can detect changes in the electron transport rate in chloroplasts when correlated with vegetation indexes. Third, hyperspectral and fluorescence tools combined with multivariate analysis can better distinguish colorful leaves and changes in variegated leaves. This study is the first to report the combined use of hyperspectroscopy and JIP test fluorescence techniques applied to *Codiaeum variegatum* (L.) A. Juss for evaluating the morphological and chemical composition in variegated leaves.

## 2. Material and Methods

### 2.1. Plant Material and Experimental Design

**Figure 1:**
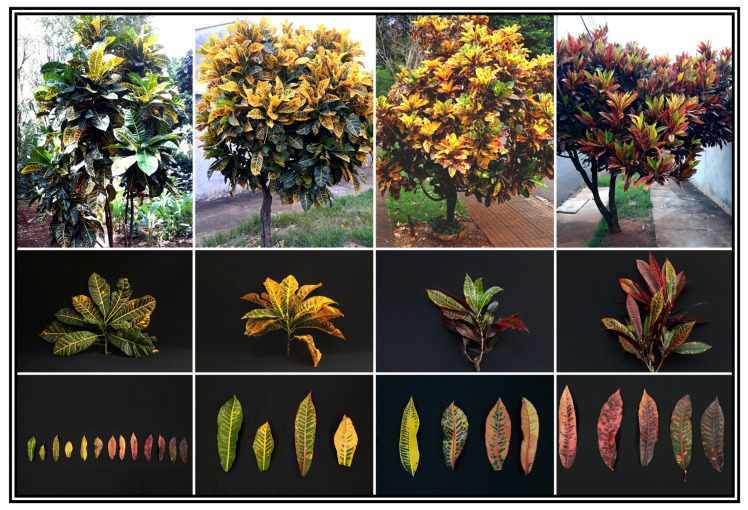
Representative image of the plants and leaves of *Codiaeum variegatum* (L.) A. Juss. The top images display the general morphological features of the plants, including the arrangement of the leaves and the accumulation of different pigments. The center images show the arrangement of leaves in phytomeres. The bottom images demonstrate the changes in pigment accumulation and variation in leaf color. From left to right, the colors depicted are green, yellow, orange, and red. The variegation of the leaves and the accumulation of different pigments can be seen from the apical to the basal region of each branch.

*Codiaeum variegatum* (L.) A. Juss (croton) plants were selected for their natural variation in leaf pigment content, color, and development from the Plant Cultivation Sector of the Botanical Garden at the State University of Maringá (Maringá, Paraná, Brazil). The plants were irrigated twice daily (8 a.m. and 6 p.m.) with sprinklers. Leaves of different ages, ontogenesis, and colors were selected from the top to the bottom of the plants (*n* = 224) for hyperspectral reflectance analysis, chlorophyll a fluorescence kinetics curves, and leaf pigment profiles. To classify the leaf color, the previous chromaticity indexes and hierarchical cluster analysis were performed across four groups: green, yellow, orange, and red leaves (Chromaticity Index Script, Wolfram Mathematica v.11.0, Champaign, IL, USA). To avoid variations in the photoperiod, environmental irradiance or dehydration that may compromise leaf comparisons when measuring hyperspectral reflectance data and fluorescence kinetics, all measurements were taken at the same time (11 a.m. to 1 p.m.) (Figure 1).

### 2.2. Spectral Data Collection

Leaf reflectance spectra were collected using a FieldSpec^®^ spectroradiometer 3 (Analytical Spectral Devices ASD Inc., Longmont, CO, USA) coupled with an ASD contact PlantProbe^®^ probe with a 10 mm diameter. The spectroradiometer had three detection sensors: one with 512 Si photodiodes capturing wavelengths from 350 to 1000 nm, and two with graduated index InGaAs photodiodes (two-stage TE cooled) capturing light from 1000 to 1800 nm and 1801 to 2500 nm. The PlantProbe^®^ leaf clip (Analytical Spectral Devices ASD Inc.; Longmont, CO, USA) was used to ensure acquiring data free of atmospheric effects. Standard white reference plates (Spectralon^®^, Labsphere Inc., Longmont, CO, USA) were used for equipment calibration and optimization. Each measurement was taken at a single point on the adaxial face of the leaves in the medial region, excluding the central rib when possible. The reflectance spectra of the leaves were obtained in the 350–2500 nm range, using a 3 nm spectral resolution for short wavelengths (350–700 nm) and 10 nm spectral resolution for longer wavelengths. The data were interpolated at 1 nm using the equipment, resulting in a total of 2151 bands. The equipment was programmed to perform 50 readings for each sample, generating an average spectral curve. A total of 224 hyperspectral leaf curves (*n* = 224) were collected with hyperspectral sensors, allowing for the variation in biophysical, pigment content and leaf color group analysis (L01–L13; see Figure 1 for variegated leaves), which were used to calculate different VIs, as described in Table S1. After the hyperspectral measurements, the corresponding leaves were placed in wipes, wrapped, and kept in the dark in a saturated humid chamber for 60 min for chlorophyll a fluorescence kinetics (ChlF) and profiling pigment measurements.

### 2.3. Fluorescence OJIP Data Collection

The chlorophyll a fluorescence induction kinetics (ChlF) data were measured using a new method-based LI-6800 IRGA (Gax Exchange System, Li-Cor Inc., Lincoln, NE, USA), and the leaves were acclimated for 60 min in a dark, humid chamber. Fluorescence curves were obtained using the following settings: a sample chamber (6 cm^2^), relative humidity (75%), CO_2_ (400 ppm), fan speed (10,000 rpm), a pulse of saturating light (625 nm) of 15,000 µmol m^−2^ s^−1^ for 1 s, dark mode at 500 Hz, and flash mode rate at 250 kHz output rate by aligning at the induction mode measure. Each point obtained for relative fluorescence intensity at 20 µs, 50 µs, 100 µs, 300 µs, 2 ms, 30 ms, and Fm_t0 − tf_ was used to calculate the JIP test parameters between 20 µs and 1 s. The curves were normalized as variable fluorescence (ΔVt), where t0 represents the initial time for fluorescence before the flash, tf denotes the final time for fluorescence after the flash, and the difference in kinetics for each OJIP phase was calculated with green leaves (L01) as a reference following Strasser et al., (2000) [29]. The five bands, ΔL (at ~20 µs), ΔK (at ~300 µs), ΔJ (at ~2 ms), ΔI (at ~10 ms), and ΔH (at ~40 ms), were calculated, resulting in 925 points of high-resolution curves. The Biolyzer software v4.0^®^ (Laboratory of Bioenergetics, University of Geneva, Geneva, Switzerland) was used to estimate the JIP test parameters associated with the electron transport chain of plants according to Table S2 and Strasser et al., (2000) [29].

### 2.4. Biophysical Parameters of Leaves

The destructive analysis of leaves was conducted to obtain the leaf blade dry weight (DW) using a forced ventilation oven for 72 h at 70 °C. The leaf area (LA) was obtained using a scanner (HP Scanjet 300, Palo Alto, CA, USA) that utilized the ImageJ software Available online: https://imagej.nih.gov/ij (accessed on 25 January 2023). Derived parameters of the specific leaf area (SLA = LA/DW) and estimation of leaf thickness (LT* = 1/(SLA × DW)) were calculated to assess physical and structural attributes associated with the vegetation index and JIP test parameters [4].

### 2.5. Profile of the Pigments Extracted

The following procedure allowed for the simultaneous quantification of total chlorophyll (Chl), carotenoids (Car), anthocyanins (AnC), and flavonoids (Flv) in leaf extracts, as described by Gitelson and Solovchenko, (2018) [31] and Falcioni et al., (2022) [32]. Briefly, the leaf segments (1 cm^2^) were ground in 2 mL tubes with a chloroform–methanol (2:1, *v*/*v*) solution in the presence of CaCO_3_. After complete extraction, distilled water (20% of the total extract volume) was added for polar and nonpolar phase separation. Extracts were centrifuged at 15,000 rpm for 5 min for full separation. All extract measurements were performed with 200 µL of a quartz glass UV 96-well microplate using a Biochrom Asys UVM-340 Microplate-Reader with ScanPlus VisibleWell^®^ software (Biochrome Ltd., Milton Road, Cambridge, UK).

#### 2.5.1. Chlorophyll and Carotenoid Quantification

The concentrations of chlorophylls *a*, *b*, *a* + *b*, and carotenoids (carotenes + xanthophylls) were measured by adding 200 µL of methanol extract to each well. The absorbance readings were performed at 470, 652, and 665 nm, and the blank sample was 100% methanol. The concentrations of chlorophylls and carotenoids (Chl*a*, Chl*b*, Chl*a* + *b*, and Car_(C+X)_) were determined using the equations defined by [33] and expressed in mg cm^−2^:

Chl*a* = 16.72 × Abs665 − 9.16 × Abs652

Chl*b* = 34.09 × Abs652 − 15.28 × Abs665

Chl*a*+*b* = Chl*a* + Chl*b*

Car(C + X) = (1000 × Abs470 − 1.63 × Chl*a* − 104.96 × Chl*b*)/221

#### 2.5.2. Flavonoid and Anthocyanin Quantification

To quantify flavonoids (Flv), they were measured in the polar phase of the methanolic extract. The upper phase, containing extrachloroplastidic pigments, was used to determine total Flv by measuring the absorbance of a microplate reader at λ358 nm with a molar absorption coefficient of ε358 = 25 mM^−1^ cm^−1^ according to Gitelson and Solovchenko (2018) [31]. Before the Flv measurement, the water–methanol phase was acidified with hydrochloric acid (HCl; final concentration of 0.1% HCl) to quantify anthocyanins (AnC) at λ530 nm using a molar absorption coefficient of ε530 = 30 mM^−1^ cm^−1^ [31].

#### 2.5.3. Total Soluble Phenolic Compound Quantification

Soluble phenol (PhC) quantification was performed based on Ragaee’s (2006) [34] method with some modifications. To initiate phenolic quantification, 150 μL methanolic extract, 70 μL Folin–Ciocalteu reagent (1 M), 140 μL Na_2_CO_3_ (3.56 M), and 850 μL deionized water were added to a 2 mL Eppendorf tube. The samples were kept in the dark for 50 min, followed by centrifugation for 120 s at 15,000 rpm. The resulting supernatant was analyzed using a quartz glass microplate reader at λ725 nm. The equivalent PhC concentration was determined using gallic acid as a reference; Ŷ = 87.651x + 1.6515; R^2^ = 0.993.

#### 2.5.4. DPPH Free Radical Scavenging Activity

To assess antioxidant activity, the free radical scavenging method using DPPH (2,2-diphenyl-1-picrylhydrazyl) was carried out as described by [5], with modifications. The DPPH solution was used at a concentration of 1 mM. The reaction started with the addition of 50 µL of the methanolic extract and 200 µL of the DPPH solution. The samples were shaken and kept in the dark for 60 min. Readings were performed in a quartz glass 96-plate microplate reader at λ515 nm [34]. The absorbances obtained were used to calculate the capacity to eliminate free radicals.

% radical scavenging activity = (1 − (Abs_DPPH_/Abs_sample_) × 100)

where:

Abs_DPPH_ = absorbance of DPPH
Abs_sample_ = absorbance DPPH after 60 min

### 2.6. Wavelength Selection Using Algorithms for Biophysical Parameters

Wavelength variable selection was performed using six algorithms: variable importance in projection (VIP), genetic algorithm (GA), sparse partial least squares regression (*s*-PLS), interval partial least squares regression (*i*-PLS), recursive partial least squares regression (*r*-PLS), and nonlinear partial least squares regression (*n*-PLS) [8]. These algorithms were used to select the most responsive wavelengths within the range of 350 to 2500 nm for assessing biophysical parameters in variegated leaves. For this purpose, MATLAB 2022a software (MathWorks, Inc., Natick, MA, USA) and PLS_Toolbox (Engenvector Research, Inc., Manson, WA, USA) were utilized for data analysis. The performance of each algorithm was evaluated based on its ability to discriminate between wavelengths and select the most responsive ones for the generated models based on weight (g leaf^−1^), leaf area (m^2^), specific leaf area (cm^2^ g^−1^), and leaf thickness (mm) (Figure S2).

### 2.7. Hyperspectral Vegetation Index for the Most Responsive Wavelength

To determine if choosing the two most responsive wavelengths by hyperspectral bands could improve the accuracy of phenomenological energy flow through excited cross-sections (CSs), we calculated all potential combinations between two spectral bands employing a normalized difference vegetation index formula (Equation (1)), following the suggestion of Crusiol et al., (2023) [35]. Each combination (involving two spectral bands under a normalized difference vegetation index formula) corresponds to one hyperspectral vegetation index (HVI), and every HVI was subsequently correlated to cross sections for phenomenological flows, evaluated with the Pearson correlation coefficient^®^ and the coefficient of determination (R^2^) using a custom-created code in the IDL language. The ground-based sensor was examined using full spectra (from 350 nm to 2500 nm). The matrices were represented in a contour map (Figure S3).

(1) $$HVI=\frac{\left(Wavelength1-Wavelength2\right)}{Wavelength1+Wavelength2)}$$

### 2.8. Statistical Analyses

#### 2.8.1. Univariate Statistical Analysis

Data were submitted to a variance homogeneity analysis via Bartlett’s test for all variables. The mean ± SD data obtained were submitted to a one-way analysis of variance (ANOVA) test, F-test (*p* < 0.001). The effects of the wavelengths on the untransformed reflectance profiles (averaged per leaf) were assessed using PERMANOVA by employing Euclidian measurements of dissimilarity using the Euclidean distance with the “vegan” package in R-Core Team 2021. Available online: https://www.r-project.org (accessed on 25 January 2023). Statistical significance was considered at *p* < 0.001 [36]. A Scott–Knott test was applied to compare data means, also at *p* < 0.001. Pearson’s correlation test was applied when applicable (*p* < 0.001). A multilevel exploratory factor analysis via hierarchical cluster methods was used to calculate tree plot-based linkage Euclidean distances. All univariate statistical analyses were performed using the Statistica 12.0^®^ software (Statsoft Inc., Tulsa, OK, USA) and Sisvar Software v.5.7 (DEX/UFLA, Lavras, MG, BRA). All graphs were prepared using the Microsoft Excel^®^ (Microsoft Office Professional 2019, Sunnyvale, CA, USA) and SigmaPlot^®^ 12.0 (Systat Inc., Santa Clara, CA, USA) software packages. In addition, the correlation analysis and correlation graphs were generated by the R software package Corrplot R-Core Team 2021. Available online: https://www.r-project.org (accessed on 25 January 2023). The pipeline models of energy fluxes through the leaf RC–CSs were created using CorelDraw 2020^®^ (Corel Corp., Ottawa, ON, Canada), based on Sitko’s model [9,30].

#### 2.8.2. Multivariate Statistical Analysis

A multivariate statistical analysis of the spectral curves (hyperspectral reflectance, chlorophyll a fluorescence kinetics, and total parameter measure) by principal component analysis (PCA) was performed using the Unscrambler X 10.4^®^ software (Camo Software, Oslo, NOR). The degree of explanation was attributed using the first two components (PC1 and PC2) prior to obtaining Fisher’s discriminant linear models. In addition, Leverage’s correction and NIPALS’s model were used to validate the residual variance. Principal component analysis graphics were obtained via a script that identified green, yellow, orange, and purple clusters (*p* < 0.90).

## 3. Results

### 3.1. Chromaticity Indexes by Color and Pigment Concentration in Leaves

In general, *Codiaeum variegatum* (L.) A. Juss. showed color and pigment variation in its variegated leaves, ranging from green to yellow to orange to red (Figure 1). For example, young leaves exhibited an accumulation of green pigments, while older leaves located at the base and away from the apical region showed a predominance of orange and red colors (Figure 1). To analyze pigment accumulation associated with biophysical, biochemical, and photochemical changes, different chromaticity indexes and single-linkage Euclidean distances were used (Figure S1).

The distribution of chlorophylls, carotenoids, anthocyanins, flavonoids, phenolic compounds, and antioxidant compounds in leaves contributed to the formation of three distinct clusters for green, yellow–orange, and red–purple, based on visual and chromaticity index plots (Figure 1 and Figure S1).

### 3.2. Biophysical Changes by Variegated Leaves

**Figure 2:**
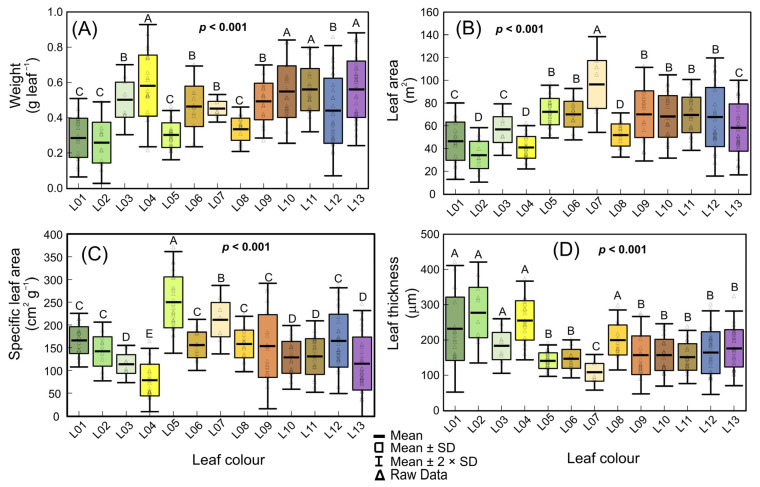
Box plot of the biophysical parameters of *Codiaeum variegatum* (L.) A. Juss due to the accumulation of pigments in variegated leaves. (**A**) Weight (g leaf^−1^). (**B**) Leaf area (m^2^). (**C**) Specific leaf area (cm^2^ g^−1^). (**D**) Estimated leaf thickness (mm). Mean ± SD and raw data were reported. Different letters over the box indicate significant differences identified from the Scott–Knott test (*p* < 0.001). (*n* = 224). L01 to L13 reference the increased green to red colors in leaves.

The boxplot and Scott–Knott tests (*p* < 0.001) displayed variegated leaf pigment changes associated with alterations in biophysical parameters (Figure 2). In general, these alterations were related to the morphological and structural organization of the leaves (Figure 2A–D; *p* < 0.001). Two to four groups were formed based on the variegated stage of development (*p* < 0.001). The younger leaves had a lower weight (W) and leaf area (LA) (0.103 to 0.875 g; 15.02 to 117.48 cm^2^) compared to those that accumulated higher levels of orange pigments (L07–13), or leaves with predominantly red pigments (Figure 2A,B). On the other hand, increases (49.6 and 125.2%) were recorded from leaves at more advanced developed stages (L07–13) or in leaves that primarily accumulated red pigments (Figure 2A,B). Consistently, the specific leaf area (SLA) decreased (59.3%) as W (weight) increased in yellow and orange leaves (r = −0.656; *p* < 0.001). However, SLA and leaf thickness decreased (44.7 and 14.6%) in leaves with higher levels of carotenoids and anthocyanins (yellow, orange, and red leaves) (Figure 2C,D).

### 3.3. Hyperspectral Reflectance in Variegated Leaves

**Figure 3:**
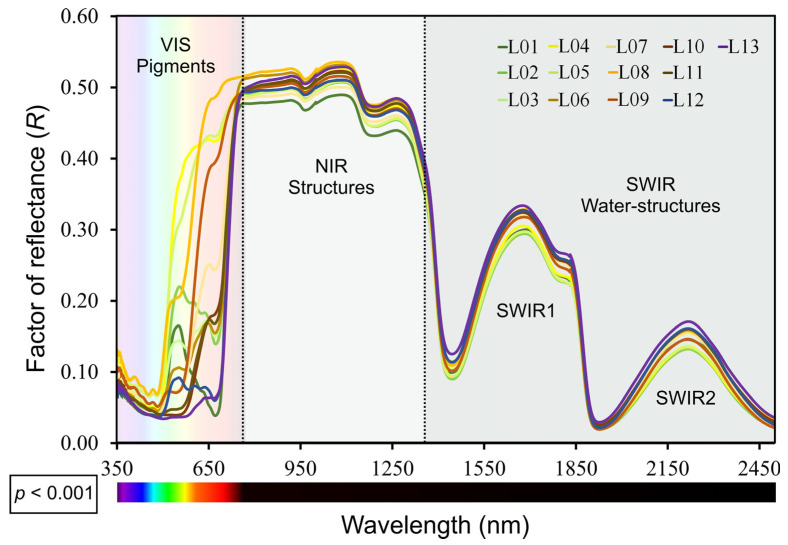
Factor of reflectance for spectral from 350 to 2500 nm in *Codiaeum variegatum* (L.) A. Juss. variegated leaves. For leaf color abbreviations (L1–13), see Figure 1. Dotted lines delimit the inflexion points of 700 and 1300 nm for VIS (visible) to NIR (near-infrared) to SWIR (shortwave infrared) bands. Mean ± SD were calculated and *p*-values from permutation analysis of variance (PERMANOVA) for the effects of leaf color on the full range (350 at 2500 nm) are reported in the bottom left corner of the panel. Each repetition represents the means of the measurements taken from green to red colors in leaves, as reported in Figure 2. Standard deviation was omitted for clarity. (*n* = 224).

The hyperspectral reflectance curves (*p* < 0.001) are linked with pigment content, concentration, biochemical properties, leaf structure, and water absorption rates across VIS–NIR–SWIR bands (Figure 3). Despite some overlap in the UV and near/medium infrared regions for the leaves with high pigment levels, spectral analysis can distinguish clusters and identify four color groups using the chromaticity index and PLS algorithm (Figure 2, Figures S1 and S2).

The leaf reflectance curves in the visible (VIS) region showed significant increases at wavelengths above 550 nm in yellow (Car and PhC) and red (AnC, Car, and PhC) leaves, despite the presence of chlorophylls (L05–L06 and L08) by specific wavelengths with *i*-PLS, *r*-PLS, and *n*-PLS algorithms (Figure S2). For example, the interaction between green and red pigments (L12) showed similar patterns to green leaves (L01, L02), with progressive reductions above 580 nm. In addition, leaves with higher concentrations of carotenoids and anthocyanins (L04, L05, L06, L07, and L08) demonstrated between 47% and 75% increments in the green and red regions (550 and 674 nm) and up to 53% increases in reflectance indexes between 500 and 800 nm (Figure 2 and Figure S2; VIS pigments). However, there were no characteristic peaks or bands between 530 and 580 nm, unlike for typical green leaves (L01) (Figure 2 and Figure S2).

In accordance with the NIR spectrum (800–1400 nm), distinct patterns among the leaves during development with advanced stages (L06–L13) showed increments compared to L01 (Figure 2 and Figure S2; NIR-structure). On the other hand, in the SWIR region (1500–2500 nm), characteristic bands of water absorption and peaks related to the structural components (1192, 1678, and 2220 nm) and phenolic compounds (1270, 1797, and 2130 nm) were observed (Figure 3 and Figure S2). This result was consistent with the *i*-PLS and *r*-PLS algorithms (Figure S2A,C,D). Furthermore, the bands close to 1500, 1600, and 1810 nm in the SWIR band can be correlated with parameters such as photochemical–photosynthetic efficiency and electron transport chain (Figure 2 and Figure S2; SWIR water structures).

### 3.4. Profiling of Pigments and Free Radical Scavenging in Variegated Leaves

**Figure 4:**
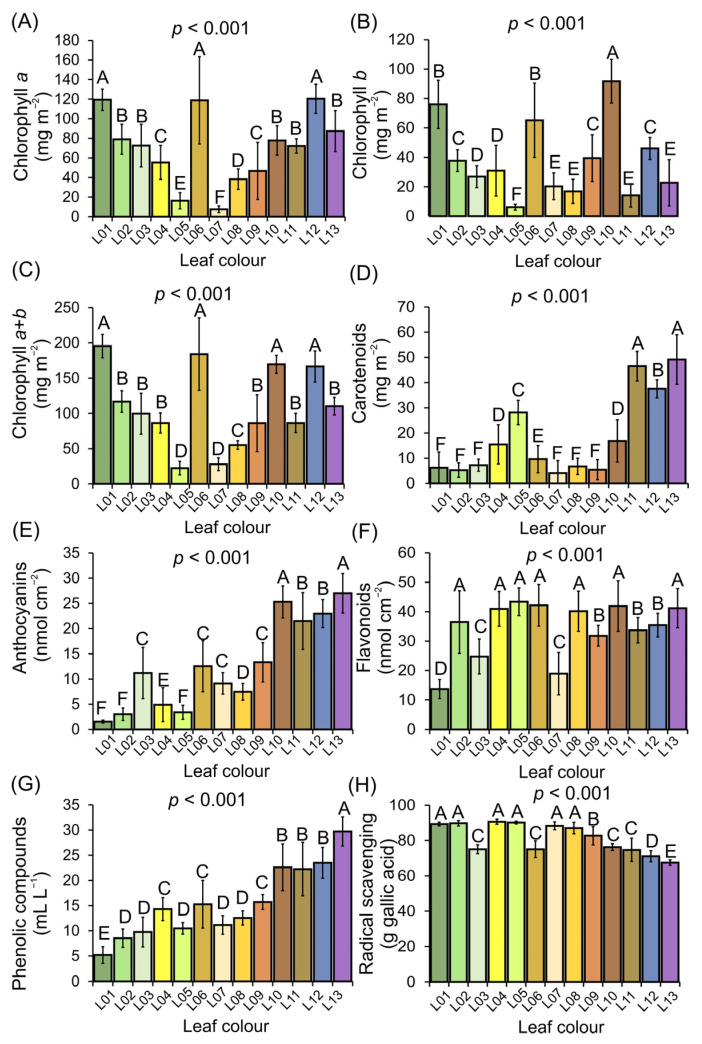
Concentration of leaf pigments in *Codiaeum variegatum* (L.) A. Juss due to green to red variegated leaves (L01–13). (**A**) Chlorophyll *a* (mg m^−2^). (**B**) Chlorophyll *b* (mg m^−2^). (**C**) Total chlorophyll (*a* + *b*) (mg m^−2^). (**D**) Carotenoids (mg m^−2^). (**E**) Anthocyanins (nmol cm^−2^). (**F**) Flavonoids (nmol cm^−2^). (**G**) Phenolic compounds (mL L^−1^). (**H**) Radical scavenging (g gallic acid). Mean ± SD. Different letters over the bars indicate statistically significant differences from the Scott–Knott test (*p* < 0.001). (*n* = 224).

A wide spectrum of pigment profiles and antioxidant capacity was quantified in variegated leaves (*p* < 0.001) (Figure 4A–H). The concentrations of chlorophyll *a* (7.64 to 119.43 mg cm^−2^) and chlorophyll *b* (6.14 to 91.65 mg cm^−2^) were independent (*p* > 0.001) of the levels of carotenoids (9.66 to 49.13 mg cm^−2^), which exhibited high concentrations in orange and red leaves, and marginally in green or yellow leaves (Figure 4A–D).

The concentration of anthocyanins increased (1.51 to 27.51 mg cm^−2^) gradually and progressed from green to red leaves (L01–L13). However, the concentration of flavonoids (13.70 to 43.79 mg cm^−2^) did not show significant correlations with the levels of anthocyanins, carotenoids, and chlorophylls (*p* > 0.05). The total soluble phenolic compounds increased (5.20 to 29.70 mg cm^−2^) progressively from yellow to orange to red leaves (L04–L13). Similarly, leaves with higher levels of carotenoids, anthocyanins, and phenolic compounds showed significant and negative correlations (r = −0.697, −0.857, −0.815) in response to free radical scavenging, indicating an increased antioxidant capacity due to variegated leaves (Figure 4E–H).

### 3.5. Vegetation Indexes and Putative Contribution for Biophysical, Biochemical, and Photochemical Parameters for Variegated Leaves

**Figure 5:**
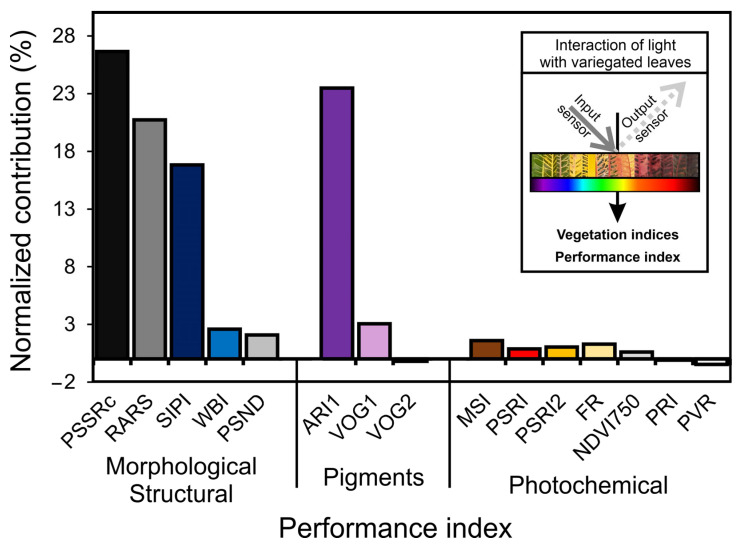
Normalized contribution of vegetation indexes (VIs) to total (100%) variability for biophysical, biochemical, and photochemical efficiency of variegated leaves. PSSRc (pigment-specific simple ratio), RARS (ratio analysis of reflectance spectra), SIPI (structurally insensitive pigment index), WBI (water band index), PSND (pigment-specific normalized difference) for biophysical parameters. ARI1 (anthocyanin reflectance index), VOG1 (Vogelmann index 1), and VOG2 (Vogelmann index 2) for biochemical parameters. MSI (moisture stress index), PSRI (plant senescence reflectance index), PSRI2 (plant senescence reflectance index 2), FR (fluorescence ratio), NDVI750 (normalized difference vegetation index ρ750), PRI (photochemical reflectance index), and PVR (normalized difference photosynthetic) for photochemical parameters. The inset displays the interaction of light with colorful leaves (arrows display the input and output sensors and generate putative relationships between the vegetation indexes), highlighting potential changes in biophysical (morphological structures), biochemical (pigments), and photochemical parameters. Each wavelength is correlated with changes in a specific vegetation index. As variegated leaves change, their interaction with light promotes changes in the most responsive vegetation indexes.

We evaluated the 15 putative most responsive VIs associated with morphological-structural, biochemical components, and photochemical parameters (Figure 5). The highest normalized contributions to the total variability performance were PSSRc (26.6%), followed by ARI1 (23.5%), RARS (20.7%), and SIPI (16.8%), which were related to specific pigments, ratios between reflectance bands, accumulation of carotenoids and anthocyanins and structural leaf thickness components, respectively. Other evaluated indexes (11) did not contribute significantly (*p* > 0.001) (Figure 5 and Table S1). The vegetation indexes for pigment variation (PSND, VOG1-2, PVR), structural contribution (CAI, NDLI, NDNI), leaf senescence indexes (PSRI1-2, CRI1-2) or even activity to change the fluorescence of chloroplast parameters, stress (WBI, MSI), and photochemical activity (PRI, FR) showed null or negative contributions (Figure 5). In addition, the NDVI750 (0.59%), widely used for vegetation characterization, did not demonstrate sufficiently consistent performance and contributions, although there were great variations among the infrared hyperspectral bands (Figure 5 and Table S1). In this sense, the inset displays the possible interaction of light with color leaves, which contributes to the selected vegetation indexes and putative contributions in estimating and monitoring evaluable to variegated leaves (Figure 5; inset).

### 3.6. OJIP Chlorophyll a Fluorescence Kinetics

**Figure 6:**
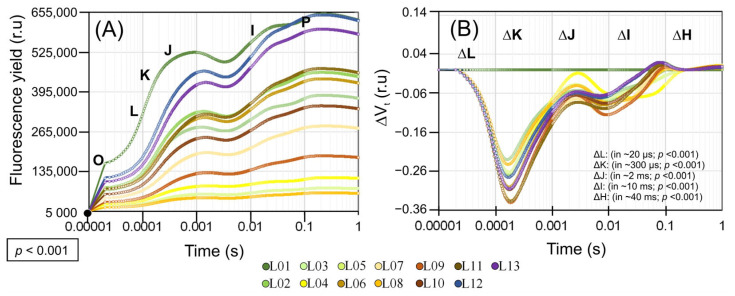
Chlorophyll a fluorescence kinetics in variegated leaves of *Codiaeum variegatum* (L.) A. Juss. Means ± SDs were calculated. (**A**) Chlorophyll a fluorescence induction kinetics. (**B**) Variable fluorescence kinetics of standardized chlorophyll (ΔVt = (Ft − F0)/Fv) − Vt control)). The ΔL, ΔK, ΔJ, ΔI, and ΔH relative changes in variable fluorescence over time, reflecting alterations in the efficiency of the light-harvesting complex (LHC), active reaction centers (RCs), and energy flow in the electron transport chain between PSII and PSI. Each repetition of variegated leaves represents the means of the measurements taken from green to red colors in leaves, as reported in Figure 2. Standard deviation was omitted for clarity. (*n* = 224). For abbreviations, see Section 2 and Table S2.

Chlorophyll a fluorescence induction kinetics, combined with the derivative JIP test parameters, allows us to evaluate information along the electron transport chain and the contribution of specific changes in photochemical and quantum efficiency of energy flow (Figure 6).

The performance and damage to the photosynthetic machinery apparatus were observed in the OJIP curves and the differences between the variable fluorescence curves associated with each leaf pigment in ΔVt. The impact of pigment variation was most significant in leaves L02–13 compared to L01 (*p* < 0.001). The progressive reduction in Chl *a*, *b*, *a* + *b*, Car, AnC, Flv, and PhC, radical scavenging was followed by decreases in ΔVt, in the four main bands in ΔL (in ~20 µs; *p* < 0.001), ΔK (in ~300 µs; *p* < 0.001), ΔJ (in ~2 ms; *p* < 0.001), ΔI (in ~10 ms; *p* < 0.001), and ΔH (in ~40 ms; *p* < 0.001).

The ΔVt kinetics in all bands (ΔL, ΔK, ΔJ, ΔI, and ΔH) were strongly negative, indicating a reduction in the efficiency of the light-harvesting complex (LHC), active RCs, and energy flow in the electron transport chain between PSII and PSI (Figure 6). This was due to decreases in chlorophyll concentrations in green and yellow leaves, and increases in anthocyanins, flavonoids, and soluble phenolics in orange and red leaves (L05–L13), which are associated with photoprotective mechanisms necessary to prevent damage to the components of the electron transport chain.

**Figure 7:**
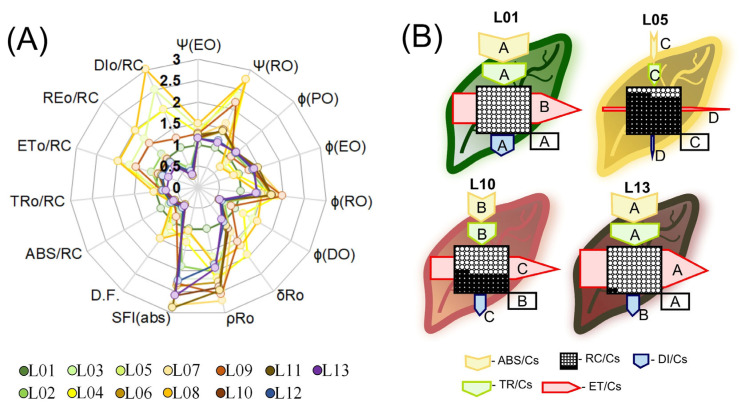
Analysis of the chlorophyll a fluorescence kinetics and pipeline flux in *Codiaeum variegatum* (L.) A. Juss due to the variegated leaves. (**A**) Radar plot indicating parameters derived from the chlorophyll a fluorescence kinetics transient (JIP test). (**B**) Pipeline leaf displayed phenomenological energy flow through excited cross sections (CSs) of *Codiaeum variegatum* (L.) A. Juss leaves in green, yellow, orange, and red representative color leaves (L01, L05, L10, L13). Yellow arrow–ABS/CS, absorption flow by approximate CS; green arrow–TR/CS, energy flow trapped by CS; red arrow–ET/CS, electron transport flow by CS; blue arrow–DI/CS, energy flow dissipated by CS; circles inscribed in squares–RC/CS, indicate the % of active/inactive reaction centers. The white circles inscribed in squares represent reduced (active) QA reaction centers, black circles represent nonreducing (inactive) QA reaction centers, and 100% of the active reaction centers responded with the highest average numbers observed in relation to the control. Arrow sizes indicate the changes in energy flows compared to the control. Each repetition was produced by taking the means of the JIP test parameters calculated and applied to representative L01, L05, L10 and L13 color leaves. Different letters over an arrow or a box indicate significant differences from the Scott–Knott test (*p* < 0.001). For abbreviations, see Section 2 and Table S2.

Absorption pigments, whether photosynthetic or not, assisted in maintaining the efficiency of the oxygen-evolving complex (OEC), reducing damage to the PSII D1 proteins, the pool of pastoquinone (PQ), plastocyanin (PC), or the ATP synthase subunits (Fo, and F1), and supported functional levels for the adequate flow energy in the transport of electrons and proton electrochemical gradient formation (ΔpH) for the synthesis of NADP and NADPH used in the stages of the Calvin–Benson cycle (Figure 6A and Figure 7A).

The OEC and D1 proteins were not affected (L10–L13) in leaves with higher levels of Flv, AnC, and PhC, as evidenced by the negative linear portion of the OJIP curve in the ΔK, ΔJ, and ΔH variation (Figure 6B). Additionally, the antioxidant activity contributed to more than 36.5% of the total free radical sequestration (Figure 6A,B).

The quantum yield of PSII (ΦPSII), thermal dissipation (ΔK), OEC decoupling (ΔJ) and the probability of electron transport from QA- to plastoquinone (ΨEo and ΦEo), in addition to the maximum quantum efficiency of PSII (ΨRo and ΦPo), increased in all cases (*p* < 0.001), except for L06 (Figure 7A). The quantum yield of energy dissipation (ΦDo) and nonphotochemical dissipation (Kn) decreased in many leaves, including ΦDo, up to 21.6%, and carotenoid accumulation in orange leaves showed increases (>51.7%) (L04, L05, and L08) (Figure 7A). The quantum yield and the likelihood of a reduction in acceptors of the final electrons on the PSI acceptor side (ρRo, ΦRo, and δRo, respectively) demonstrated significant increases (~86.3%) in response to L01 (Figure 7A). This was also corroborated by the observations of the ΔI and ΔH bands, which prevented damage to energy transfer by reducing the concentration of carotenoids, anthocyanins, flavonoids, and phenolic compounds in the variegated leaves (Figure 6).

### 3.7. Target Modeling of the Fluorescence Kinetics of Phenomenological Energy Fluxes in Variegated Leaves

Phenomenological models of energy flow by cross sections (CSs) are demonstrated in Figure 7. Due to the fluorescence kinetics information following the function of the hyperspectral patterns and targeting pigments for profiling, it was possible to obtain a quantitative estimate of the photosystem’s efficiency levels and photoprotection/extinction mechanisms present in the electron transport chain between PSII and PSI in leaves with different colors (Figure 7A).

The decrease or degradation in Chl, Car, AnC, Flv, and PhC content reduced the energy flow, as well as scavenging activity, compared to green, orange–green, and red leaves (Figure 7). Additionally, the reduction in Car levels promoted severe damage to the antenna complex (ABS) and reaction centers (RCs) of the photosystems. This contributes to the reduction of the required energy intake for the electron transport chain and the formation of reducing power in the form of NADPH and ATP (Figure 7B). The fraction of energy that would be available for absorption by a cross section of the leaves (ABS/CS), energy capture (TR/CS), and electron transport flow (ET/CS) decreased (>79.3%) in relation to green leaves (L01–L03). However, leaves that accumulated AnC, Flv, and PhC exhibited increased antioxidant activity and reduced effects on energy flow (Figure 7B, sizes of arrows and active RCs). Based on the models (Figure 7B), the electron transport chain appears to be on the verge of imminent collapse (L05–L07) or experiencing a more moderate collapse (L09–L10), taking into account the relative energy flow from RCs to ET/CS (Figure 7B). In addition, the wavelength for VIS contributes to these changes (Figure S3).

The models also demonstrate that energy dissipation (DI/CS), even with a reduced accumulation of carotenoids, can be directed to other pigments considered to be accessories (Figure 7B, arrows). Plants with reduced levels of Car, AnC, and Flv, in addition to impairing the efficiency of electron transport and energy or thermal dissipation, showed signs of chronic photoinhibition and reduced the performance of PSII and PSI (L05 and L08). Furthermore, our data demonstrated that even a minor accumulation of AnC, Flv, and PhC contributed to an increase in energy dissipation and a reduction in free radical scavenging activity, according to L10. Thus, in response to pigment biosynthesis and greater antioxidant capacity, it could be demonstrated that photochemistry efficiency was only slightly compromised, following a specific interaction with each wavelength (Figure S3). According to ABS/CS, TR/CS data, DI/CS, and ET/CS in orange and red leaves, although these leaves exhibited fewer open reaction centers (RCs) and an oxidized QA^−^ in relation to L01, they were photosynthetically functional (*p* < 0.001) (Figure 7B and Figure S3; L10).

### 3.8. Hyperspectral Reflectance, Fluorescence Kinetics, Variables Total Performance and Pearson’s Correlation Coefficients Associated with Biophysical, Biochemical and Photochemical Parameters

**Figure 8:**
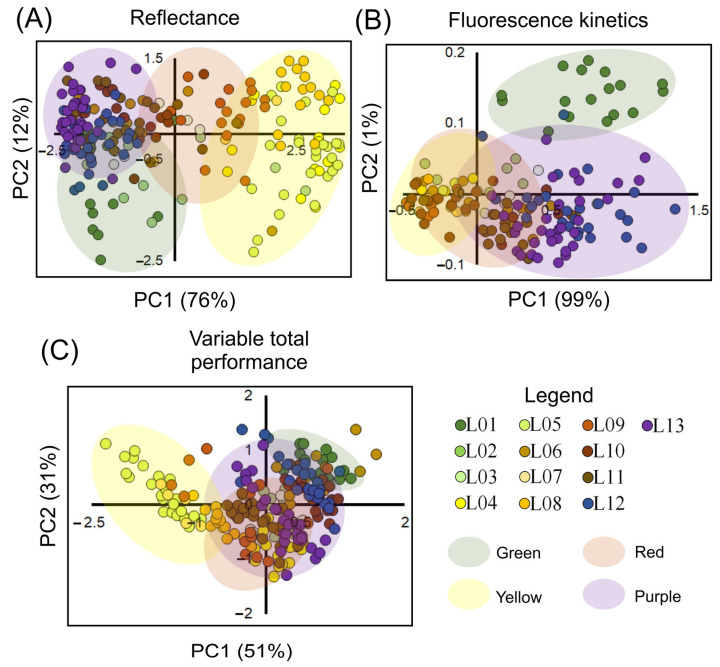
Principal component analysis (PC) of the *Codiaeum variegatum* (L.) A. Juss due to pigment accumulation in variegated leaves. (**A**) Hyperspectral reflectance data. (**B**) Chlorophyll a induction kinetics (fluorescence kinetics). (**C**) Variable total performance between reflectance and fluorescence kinetics data. Clustering color leaves are displayed as green, yellow, red, and purple circles.

The principal component analysis (PCA) indicates a greater number of clusters when compared to other statistical tools previously used in classification (Figure 8). For the reflectance data, the first two components, PC1 and PC2 (accounting for 76% and 12% of the data variability, respectively), explained 88% of the total data variability in the curves from 350 to 2500 nm (Figure 8A). Similarly, for fluorescence kinetics curves, PC1 and PC2, which accounted for 99% and 1% of the data variability, respectively, explained 100% of the data variability (Figure 8B). When morphological and anatomical variables, pigment concentrations, vegetation indexes, and photochemical fluorescence were combined, they contributed to 82% of the total data set, with PC1 and PC2 accounting for 51% and 31% (Figure 8C), respectively.

**Figure 9:**
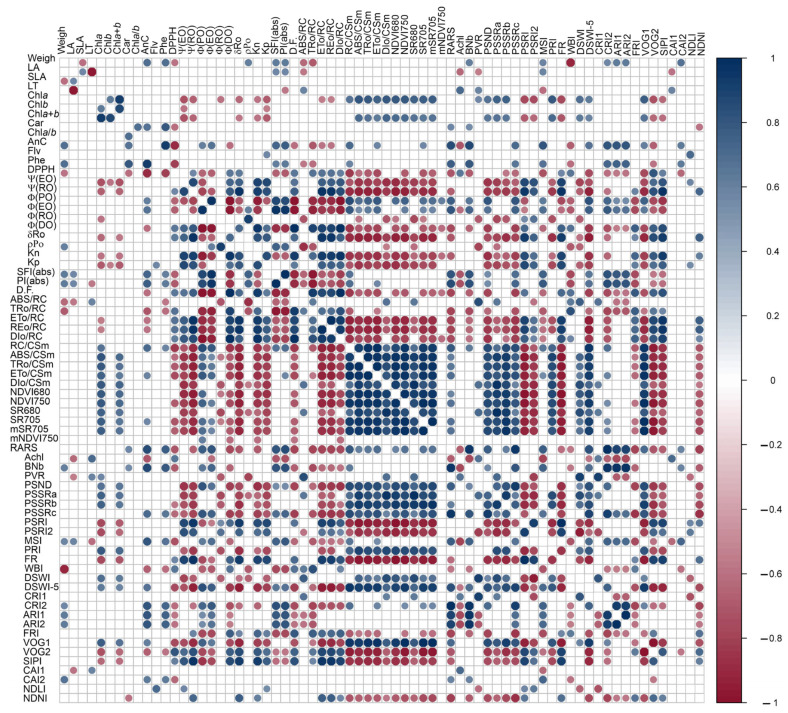
Pearson’s correlation matrix (*p* < 0.001). Abbreviations are described in the Materials and Methods section and Tables S1 and S2.

Figure 9 shows the correlations between the concentrations of chlorophylls and NDVI750 indexes PSRI1-2, VOG1-2, PRI, FR, and NDVI680 (r ≥ 0.559; *p* < 0.001). Anthocyanins were found to be correlated with ΦEo, SFI(abs), PI(abs) vs. NDVI750, RARS, ARI1, VOG1-2, Achl, BNb, CRI2, CAI2, and DSWI-5 (r ≥ 0.532; *p* < 0.001). It is worth noting that several variables derived from the JIP test curves correlated with vegetation indexes (VIs) as well. For example, PSRI was correlated with ΨRo, δDo, and Kp (r = 0.587, 0.659, 0.667). FR was correlated with δDo and Kp (r = 0.657, 0.779), and NDVI680 was correlated with δDo and Kp (r = −0.649, −0.771). BNb was correlated with SFI(abs) and PI(abs) (r = 0.617, 0.657), PRI was correlated with Kp (r = −0.654), and PSRI was correlated with δDo and Kp (r = 0.659, 0.764). CRI2 was correlated with PI(abs) (r = 0.588), ARI1 was correlated with SFI(abs) and PI(abs) (r = 0.584, 0.632), VOG1 was correlated with Kp (r = −0.673), and DSWI-5 was correlated with ΨEo, ΨDo, KP, SFI(abs), and PI(abs) (r = 0.556, −0.553, −0.739, 0.501, 0.539).

Moreover, some vegetation indexes (VIs) contributed more significantly to the correlations between pigments and antioxidant mechanisms and parameters derived from fluorescence kinetics curves for thermal dissipation (Figure 9). VIs such as RARS, ARI1, SIPI, and PSSRc showed stronger correlations (r ≥ 0.53; *p* < 0.001) with nonchloroplastidic photoprotectors (AnC, PhC). However, carotenoids demonstrated poor correlations with other derivatives of the JIP test when compared to VIs such as CRI1-2, VOG1-2, and PRI. The PRI displayed a strong correlation with Chls and Kp (>±0.651), and indirectly with other VIs, including NDVI750, NDVI680, PSRI1-2, VOG1-2, SR680, SR705, PSSRa, PSSRb, and DSWI-5 (>±0.723). These VIs showed an indirect relationship with pigment levels and photochemical efficiency in *Codiaeum variegatum* leaves (Figure 8 and Figure 9).

## 4. Discussion

In this study, alternative methods such as hyperspectral reflectance and fluorescence kinetics curves were combined with multivariate statistical analyses to increase the understanding of changes in the electron transport rate and energy flux in chloroplasts [8,27]. The results showed the highest correlation between JIP test data and biophysical, biochemical, and photochemical changes in croton leaves, with many associations found between vegetation indexes (VIs). The most responsive VIs, including PSSRc, ARI1, RARS, and SIPI, were strongly correlated with pigment levels, while others, including MSI, PVR, PRI, FR, and NDVI, were linked to the photochemical components of photosynthesis (Figure 4 and Figure 9). However, the total relative contribution was low (Figure 5). Nevertheless, many VIs showed strong correlations (r ≥ ±0.651) with parameters derived from the JIP test (Figure 6, Figure 7, Figure 8 and Figure 9 and Figures S1–S3).

Our findings suggest that structural and biochemical changes in variegated leaves linked to the accumulation of pigments can be detected through hyperspectral reflectance curves, absorbance curves, [8,27] photosynthetic data, and metabolomics [37]. Other techniques, such as photosystem proteins [38], spectroscopy [39], antioxidant activity [40], heavy metal contamination [30], primary and secondary metabolites [12], gene expression, and molecular markers [41], can also be used to evaluate changes in leaf pigments and variegation [42]. By using these techniques, new insights into the relationship between variegated leaves, pigments, and structural and biochemical changes in croton leaves can be gained, as well as in other plants. Overall, our study confirmed three initial hypotheses, which are discussed in more detail in the following sections.

### 4.1. The Effect of Morphological, Physiological, and Anatomical Attributes on the Vegetation Indexes

The use of vegetation indexes (VIs) has been shown to be an effective way of identifying consistent morphological and anatomical changes in plants [6]. According to, Falcioni et al., (2020) [5] and Fernandes et al., (2020) [6], these changes are often related to the biosynthesis and degradation of pigments, such as chlorophylls, carotenoids, anthocyanins, phycobiliproteins, and flavonoids, as well as total antioxidants, which are influenced by many structural components and cellular organelles, such chloroplasts, vacuoles, and pastoglobules during leaf development [5]. Furthermore, the distribution of pigments in the leaf profile can be affected by changes in specific leaf area (SLA) and leaf thickness, which can result in different VIs [5,6]. Thus, the use of VIs allows for the non-destructive evaluation of changes in leaf pigments, as reported in Coast et al., (2019) [43], and other tissues in plants, providing a useful tool for understanding the development responses and environmental interaction factors such as temperature, photoperiod, radiance, and light qualities [44,45,46].

Accordingly, hyperspectral changes observed in the near-infrared and shortwave-infrared bands are attributed mainly to the organization and structure of cellular components, such as cell wall components (cellulose, lignin, nitrogen), water content, and air cell interfaces, rather than pigments [5,13]. In this way, these changes may not always correlate with classic VIs, such as NDVI, VOG, PSI, and PRI, but are observed at the ontogenetic development level and influenced by environmental factors such as irradiance intensity, quality, and duration [21,43,47]. In accordance with Fine et al., (2021) [37] and Sinanoglou et al., (2018) [40], vegetation indexes (VIs) such as PSSRc, ARI1, RARS, and SIPI may be more effective in identifying changes in the structural components and cell organelles during leaf development in light qualities or variegated leaves.

Furthermore, leaf internal structure, such as mesophyll cell volume, can influence energy absorptivity and flow within the leaf [3,5]. Other studies have demonstrated energy dissipation components, nonphotosynthetic pigments, thermal influences, efficient water use, and leaf water content [13,21,48,49]. Therefore, these components contribute to changes in VIs across the entire spectral range (350–2500 nm).

### 4.2. The JIP Test Parameters Indicate Dissipation to the Thermally Based Vegetation Indexes

The JIP test demonstrated a correlation between reduced levels of carotenes and xanthophylls and photosynthetic performance indexes (Kp, PI(abs), SFI(abs), and D.F. [50,51]. This suggests the existence of alternative mechanisms for photoprotection and a decline in the electron transport rate, with significant implications for both the biochemical and photochemical processes of photosynthesis [50,51]. Moreover, employing hyperspectral and fluorescence technique measurements can further reinforce these findings.

A strong correlation was observed between the PRI and photochemical indexes (OJIP variables) and environmental changes on a global scale, despite the lack of correlation with Car [52]. Following Bag et al., (2020) [50] and Jin et al., (2020) [51], there are two potential explanations for this finding. Firstly, from a statistical perspective, the high resolution of reflectance and fluorescence kinetics factors enables a positive association between each generated point and the VIS. This remains true even when the VIs may not be sufficiently robust for detecting changes in multispectral analysis or hierarchical cluster analysis [53]. Second, from a physiological perspective, nonphotochemical processes, such as the presence of accessory pigments (AnC, PhC, Flv, or antioxidants), can influence the energetic dissipation mechanisms (Figure 4, Figure 5 and Figure 7), which impacts the efficiency of certain indexes not correlated with photochemical efficiency, aligning with the initial hypotheses. For instance, the presence of pigments that enable energy dissipation or antioxidant activity (AnC, PhC, or scavenging) was found to be highly correlated with the PRI, PSRI, CR1, VOG1-2, and MSI (Figure 5, Figure 8 and Figure 9).

Our findings suggest that the PRI, PSRI, and VOG1-2 variables of VIS-VIs are substantially influenced by orange and red pigment accumulation and photochemical activity in variegated leaves. Conversely, the FRI and NDLI are associated with pigments and energy absorption by phenomenological components, but do not significantly impact photochemical efficiency. Additionally, a strong correlation was observed between anthocyanin and flavonoids or other phenolic compound levels with ARI1, but not with CRI1, FRI, or FR (Figure 5, Figure 7B, Figure 9, Figure S2 and S3). Therefore, our first hypothesis has been confirmed, as many vegetation indexes can be utilized to evaluate, separate, and correlate changes in orange–red–purple variegated leaves that have not been discussed in previous research.

### 4.3. PRI and PSSRc Indexes Are Strongly Associated with VIS–NIR–SWIR Bands

The application of the PRI and PSSRc indexes in analyzing the hyperspectral reflectance curves of variegated leaves or large areas of uneven vegetation has been an effective tool in understanding rapid changes in leaf development and pigment accumulation patterns [5,13,43]. According to Braga et al., (2021) [13], PSSRc indexes are strongly associated with the variations and interactions of NIR–VIS bands, which contribute to the improved classification and use of vegetation indexes (VIs) [1,2,3]. However, it is important to note that VIs are associated with reflectance characteristics and linked with the surface of biomaterials, which can lead to a loss of information related to pigment absorbance patterns at the leaf level [3,5]. Thus, VIs such as NDVI, FR, or even PRI were not highly correlated with the broad spectrum of pigments in this study (Figure 2, Figure 5 and Figure 9), while PSSRc made a significant contribution (26.6%), contrasting with the data found by Onoda et al., (2017) [1].

According Falcioni et al., (2020) [5] and Coast et al., (2019) [43], the factor of reflectance (*R*) in detecting changes in plant physiology has been shown to be effective in previous studies [6,13,48] (Figure 3 and Figure 8). However, the impact of surface interactions with the factor of reflectance (*R*) and leaf components such as waxes, stomata, epicuticular layers, or trichomes can affect the accuracy of vegetation indexes (VIs) [5]. For example, the Gitelson group has developed VIs that take colorimetric variations into consideration. Furthermore, advances in chemometric data analysis and modeling to address specific plant biology should be solved by applied VIs and spectroscopy techniques. These indexes have been found to be effective in analyzing changes in plant physiology and are reported in our data. In this way, the NIR–SWIR and VIS regions are particularly useful for discerning information about the PSII and PSI for increased or decreased photosynthesis rates (Figure 5, Figures S2 and S3). Therefore, future studies could explore the potential of these indexes in assessing the impact of biotic and abiotic factors, such as diseases and drought, and other changes in photosynthetic dynamics within plant physiology research.

### 4.4. PSSRc Photosynthetic Apparatus Could Be Measured Using Fluorescence Techniques Based on Vegetation Indexes in Variegated Leaves

The JIP test curves are effective in tracking the absorption and release of saturating light as photochemical and nonphotochemical processes through the QA redox principle [54]. These curves can be more effective when combined with ontogenetic standards and leaf pigment concentrations and contents for a better understanding of data viability [5]. Our analysis shows that the OJIP curves are the most effective for measuring the PSSRc aligned with the photosynthetic apparatus in colorful leaves (Figure 5, Figure 6, Figure 7 and Figure S3) compared to the sum of individual reflectance parameters and fluorescence spectroscopy. The clusters observed in response to variated leaves in our study reveal the role of various pigment classes and concentrations in the biophysical and photochemical interactions between PSII and PSI [20,41]. This is crucial, as the dissipation pathways through NPQ, qL, or qE quenching and photoprotection mechanisms in chloroplasts differentiate photochemical and nonphotochemical mechanisms resulting from pigment absorption (Figure 7B) [21].

For example, the discussion of the quenching transition (qT) can be mediated, resulting in the formation of energy dissipating supercomplexes (LHCII-LHCI-PSII-PSI) [50,55], leading to fluorescence indexes correlating with other indexes such as SIPI, PRI, and FR, as reported in Figure 5, Figure 6, Figure 7, Figure 8 and Figure 9. In the future, further research should focus on optimizing and applying OJIP curve align optical spectroscopy, Raman microspectroscopy analysis, and other techniques to improve the accurate measurement of the PSSRc photosynthetic apparatus in various vegetation and other species.

In addition, the importance of fluorescence indexes such as the SIPI and FR in determining the photochemical parameters of photosynthesis, is highlighted. In accordance with Wang et al., (2017) [56] and Bag et al., (2020) [50], these vegetation indexes showed a better correlation with photochemical parameters than with pigment concentration (Figure 4 and Figure 9), which supports previous research suggesting a decrease in fluorescence yield due to changes in thylakoid stacking and the yellowing of leaves [50,56].

On the other hand, other indexes, such as CRI1-2, which are commonly associated with carotene contents, showed weak correlations with a wide range of green-to-red leaves (Figure 1, Figure 5 and Figure 9). To improve accuracy, Gitelson and Solovchenko (2018) [31] and Falcioni et al., (2020) [5] recommend using hyperspectral absorbance curves as additional measures in combination with fluorescence and hyperspectral tools, to better understand the optical changes in leaves. When considered in groups, these optical spectroscopy methods are promoted as alternative methods to enhance photochemical monitoring in tobacco, lettuce and many native species [5,6,20].

The use of the JIP test and fluorescence indexes in studying the photosynthetic processes in leaves offers several advantages [26,27,57,58]. The JIP test is capable of identifying changes in the components of ABS/CS, TR/CS, and ET/CS that correlated with the leaf pigment concentration, primarily chlorophyll and other accessory pigments (Figure 8 and Figure S3). It also allows for the measurement of thermal and dissipative effects, encompassing both photochemical and nonphotochemical effects [38,43,50]. For example, fluorescence indexes such as SIPI and FR can provide information on the electron transport chain and its efficiency, as well as important JIP test derivatives, such as ΦPSII, ΨEo, ΨRo, δDo, PI(abs), SFI(abs), and D.F. [38,43,50].

The combination of the JIP test and fluorescence indexes facilitates a more comprehensive understanding of the photochemical processes in leaves with varying pigments, such as Chl, Car, AnC, Flv, and PhC [26,59]. In this regard, it is valuable for monitoring changes in vegetation health and productivity at different scales, including field, regional, and global scales [18,60]. Future research should concentrate on employing these tools in this context. Additionally, our second and third hypotheses were confirmed, demonstrating correlations between fluorescence kinetics and VIs, as well as alterations in the electron transport chain of chloroplast membranes. As a result, hyperspectroscopy and fluorescence tools, combined with multivariate analysis (Figure 8C), exhibited high accuracy and precision in distinguishing these changes.

### 4.5. Novel Perspective of Hyperspectral–Fluorescence Techniques

Hyperspectral and fluorescence tools have been previously used in studies with other plant species, such as tobacco, wheat, and coffee, to develop new indexes linking fluorescence parameters with the electron transport chain [19,61,62,63]. Our study on *C. variegatum* offers two novel perspectives using these tools. Firstly, fluorescence kinetics can be effectively combined with hyperspectral tools to understand a plant’s photochemical and photosynthetic capacity (Figure 8 and Figure 9). Second, the separation of different clusters enables the classification of at least 13 distinct variegated leaves with varying pigment concentration and contents [38,43,50]. Accordingly, the results should support the integration of the JIP test into digital fluorescence airborne cameras and linked vegetation indexes in mapping large, nonhomogeneous areas to detect changes in variegated leaves [50,56]. This allows for the evaluation of photochemical components, differentiation between them, and the monitoring of plant productivity in extensive crop fields, native species, and plant ecosystems in real time.

The combination of hyperspectral and fluorescence techniques has proven to be rapid, simple, and cost-effective (Figure 8C and Figure 9). This approach has led to the validation of several indexes related to different pigment classes, through pigment profiling and fluorescence kinetics linked to wavelength HVI algorithms (Figure S3). Therefore, indexes such as PSSRc, PSRI, SIPI, and ARI have been found to be good predictors in the VIS–NIR–SWIR range, as demonstrated in Figure 5 and Table S1, and should be used to identify unrelated associations. These indexes not only predict pigment concentrations, but also offer valuable insights into photosystems and vegetation dynamics. They account for differences in indexes such as reflectance factors (*R*) and the effects of potential environmental factors on plant ecosystems, ranging from organelles to extensive terrestrial environments [64,65]. However, it is crucial to conduct more in-depth evaluations of these VIs when analyzing data collected from plants using optical spectroscopy and remote sensing techniques.

## 5. Conclusions

In summary, our study demonstrates the utility of integrating hyperspectral and fluorescence techniques associate with pigment profiling and vegetation indexes for understanding the complex biophysical, biochemical, and physiological changes of *Codiaeum variegatum* (L.) A. Juss in variegated leaves. Our findings reveal a novel approach in this field, as well as enable the classification of different stages of color leaves using the JIP test that can associate them with vegetation indexes. Overall, the integration of hyperspectral and fluorescence tools has improved our knowledge of plant physiology and holds significant potential for future research in plant ecology, optical spectroscopy, and remote sensing applications.

## Data Availability

The data presented in this study are available in Supplementary Materials.

## Supplementary Materials

The following supporting information can be downloaded at: https://www.mdpi.com/article/10.3390/biology12050704/s1, Figure S1: Chromaticity index obtained using single linkage Euclidean distances and the formation of three clusters associated with variegated leaves that are green, yellow, and red-purple; Figure S2: Selected most responsive variables among the wavelengths of 350–2500 nm by VIP, GA, *s*-PLS, *i*-PLS, *r*-PLS, *n*-PLS algorithms for variegated leaves. (A) Weight (g). (B) Leaf area (m^2^). (C) Specific leaf area (cm^2^ g^−1^). (D) Estimated leaf thickness (µm); Figure S3: Count plot map of coefficient of correlation (R^2^) from the linear regression between phenomenological energy flow through excited cross-sections (CSs) of *Codiaeum variegatum* (L.) A. Juss leaves and wavelengths1 vs. wavelength2 for 350 to 2500 nm. (A) RC/CS, indicate the % of active/inactive reaction centers. (B) ABS/CS, absorption flow by approximate CS; (C) TR/CS, energy flow trapped by CS. (D) ET/CS, electron transport flow by CS. (E) DI/CS, energy flow dissipated by CS. Dark blue to red displayed increased associations; Table S1: Biophysical, biochemical and photochemical parameters-based efficiency-related for vegetation indices (VIs); Table S2: Parameter derivation of OJIP chlorophyll a fluorescence kinetics induction. References [23,29,66,67,68,69,70,71,72,73,74,75,76,77] are cited in the Supplementary Materials.
