# Supplementary material for: Biophysical, Biochemical, and Photochemical Analyses Using Reflectance Hyperspectroscopy and Chlorophyll a Fluorescence Kinetics in Variegated Leaves
Source: Biology (Basel). 2023 May 11;12(5):704. doi: 10.3390/biology12050704 (PMC10215320; doi:10.3390/biology12050704)
Supplement: Supplementary file 1 [file biology-12-00704-s001.zip › biology-2287535-supplementary.pdf]

biology

MDPI

Supplementary file

Biophysical, Biochemical, and Photochemical Analyses Using Reflectance Hyperspectroscopy and Chlorophyll a Fluorescence Kinetics in Variegated Leaves

Renan Falcioni [1,*], Werner Camargos Antunes [1], José A. M. Demattê [2] and Marcos Rafael Nanni [1]

1 Department of Agronomy, State University of Maringá, Av. Colombo, 5790, Maringá 87020-900, Paraná, Brazil; wcantunes@uem.br (W.C.A.); mrnanni@uem.br (M.R.N.)
2 Department of Soil Science, Luiz de Queiroz College of Agriculture, University of São Paulo, Av. Pádua Dias, 11, Piracicaba 13418-260, São Paulo, Brazil; jamdemat@usp.br
* Correspondence: renanfalcioni@gmail.com; Tel.: +55-44-3011-8940

Table S1. Biophysical, biochemical and photochemical parameters-based efficiency-related for vegetation indexes (VIs).

Index	Equation	Reference
NDVI$_{680}$ = Normalized Difference Vegetation Index ρ680	$(R_{800}-R_{680})/(R_{800}+R_{680})$	[23,29,66]
NDVI$_{750}$ = Normalized Difference Vegetation Index ρ750	$(R_{750}-R_{705})/(R_{750}+R_{705})$	
SR$_{680}$ = Simple Ratio Index ρ680	(R_{800}/R_{680})	[66]
SR$_{705}$ = Simple Ratio Index ρ705	$(R_{750})/(R_{705})$	
mSR$_{705}$ = Modified Normalized Simple Ratio ρ705	$(R_{750}-R_{445})/(R_{705}+R_{445})$	[66]
mNDVI$_{750}$ = Modified Normalized Difference Vegetation Index ρ750	$(R_{750}-R_{705})/(R_{750}+R_{705}-2\times R_{445})$	
RARS = Ratio Analysis of Reflectance Spectra	$(R_{746})/(R_{513})$	[66]
Achl = Absorption of Chlorophyll Index	$(R_{550})/(R_{500})$	
BNb = Index for Chlorophyll Content	$(R_{800})/(R_{550})$	[66]
PVR = Normalized Difference Photosynthetic	$(R_{550}-R_{650})/(R_{550}+R_{650})$	
PSND = Pigment Specific Normalized Difference	$(R_{800}-R_{470})/(R_{800}+R_{470})$	[66]
PSSRa = Pigment Specific Simple Ratio Chl *a*	$(R_{800})/(R_{680})$	
PSSRb = Pigment Specific Simple Ratio Chl *b*	$(R_{800})/(R_{635})$	[67]
PSSRc = Pigment-specific Simple Ratio	$(R_{800})/(R_{500})$	
PSRI = Plant Senescence Reflectance Index	$(R_{680}-R_{500})/(R_{750})$	[68]
PSRI2 = Plant Senescence Reflectance Index 2	$(R_{672})/(R_{550}+R_{708})$	
MSI = Moisture Stress Index	(R_{1650}/R_{830})	[68]
PRI = Photochemical Reflectance Index	$(R_{530}-R_{570})/(R_{530}+R_{570})$	
FR = Fluorescence Ratio	$(R_{690})/(R_{740})$	[69-73]
WBI = Water Band Index	$(R_{900})/(R_{970})$	
DSWI = Disease-Water Stress Index	$(R_{802}+R_{547})/(R_{1657}+R_{682})$	[70]
DSWI-5 = Disease-Water Stress Index 5	$(R_{800}-R_{550})/(R_{1660}+R_{680})$	
CRI1 = Carotenoid Reflectance Index 1	$(1/R_{510}) - (1/R_{550})$	[70]
CRI2 = Carotenoid Reflectance Index 2	$(1/R_{510}) - (1/R_{700})$	
ARI1 = Anthocyanin Reflectance Index	$(1/R_{550}) - (1/R_{700})$	[70]

ARI2 = Anthocyanin Reflectance Index 2	$R_{800} \times ((1/R_{550}) - (1/R_{700}))$	
FRI = Flavonol Reflectance Index	$R_{800} \times ((1/R_{410}) - (1/R_{460}))$	[71]
VOG1 = Vogelmann Index 1	$(R_{740})/(R_{720})$	
VOG2 = Vogelmann Index 2	$(R_{734}-R_{747})/(R_{715}+R_{726})$	[70]
SIPI = Structurally Insensitive Pigment Index	$(R_{800}-R_{445})/(R_{800}-R_{680})$	
CAI1 = Cellulose Absorption Index 1	$100 \times (0.5(R_{2030}+R_{2210}) - R_{2100}))$	[71]
CAI2 = Cellulose Absorption Index 2	$0.5 \times (R_{2020}+R_{2220}) - R_{2100}))$	
NDLI = Normalized Difference Lignin Index	$[\log(1/R_{1754}) - \log(1/R_{1680})]/[\log(1/R_{1754})+\log(1/R_{1680})]$	[72–76]
NDNI = Normalized Difference Nitrogen Index	$[\log(1/R_{1510}) - \log(1/R_{1680})]/[\log(1/R_{1510})+\log(1/R_{1680})]$	[77]

Table S2. Parameter derivation of OJIP chlorophyll *a* fluorescence kinetics induction.

**Information selected from the fast OJIP fluorescence induction (data necessary for the calculation of the so-called JIP test parameters - LiCor-6800-Multiphase Flash™ Fluorometer - induction curves)**	
**Fluorescence parameters**	
$F_O = F_{20\ \mu s}$	First reliable fluorescence value after the onset of actinic illumination; used as initial value of the fluorescence
$F_{50\ \mu s}$	Fluorescence value at 50 μs
$F_{100\ \mu s}$	Fluorescence value at 100 μs (L-level)
$F_{300\ \mu s}$	Fluorescence value at 300 μs (K-level)
$F_J \equiv F_{2\ ms}$	Fluorescence value at 2 ms (J-level)
$F_I \equiv F_{30\ ms}$	Fluorescence value at 30 ms (I-level)
$F_P \equiv F_M$	Fluorescence value at the peak of OJIP curve; maximum value under saturating light
$t_{Fmax} \equiv t_{FM}$	Time to reach the maximum fluorescence value F_M
Area	Area between OJIP curve and the line $F = F_M$; also total complementary area (from time 0 to t_{Fmax}) over the fluorescence induction curve is a measure of the number of quanta not emitted as fluorescence as a consequence of the photochemistry during the induction phase
**Technical fluorescence parameters**	
F_O/F_M	Expresses the ratio fluorescence in leaves acclimated in dark and maximum fluorescence after saturation pulse light (13,000 μmol m^{-2} s^{-1})
F_V/F_O	Expresses the ratio between variable (F_M-F_O) fluorescence and initial fluorescence (F_O)
$V_t \equiv F_O/F_V \equiv (F_t - F_O)/(F_M - F_O)$	Relative variable fluorescence
$M_O \equiv (dV/dt)_O \equiv (\Delta V/\Delta t)_O$	Expresses the rate of the RC's closure
$(dVG/dt)_O \equiv (\Delta VG/\Delta t)_O$	Expresses the excitation energy transfer between the RCs
N	Expresses how many time Q_A has been reduced in the time span from time 0 (t=0) to t_{FM}
$S_M \equiv Area/F_V$	Normalized area (assumed proportional to the number of reduction and oxidation of one Q_A^- molecule during the fast OJIP transient, and therefore related to the number of

	electron carriers per electron transport chain. **This a measure of the energy needed to close all reaction centers**
V_J	Variable fluorescence 2 ms (J-value)
V_I	Variable fluorescence 30 ms (I-value)
**Definitions of energy fluxes at joules (J)**	
$J^{ABS} = J^{TR} + J^{DI}$	Rate of photons absorption by total PSII antenna-denoted *as absorbed photon flux*
J^{TRo}	Maximum (initial) trapped flux
J^{ETo}	Electron transport flux $\mathbf{Q_A}$ to $\mathbf{Q_B}$
J^{DI}	Rate of energy dissipation in all the PSIIs, in processes other than trapping – denoted as *dissipated energy flux*
J^{REo}	Electron transport flux until PSI acceptors (defined at *t=30 ms*, corresponding to the 1-level)
**Quantum yields, efficiencies and rates/probabilities**	
$\psi_{(EO)} \equiv \psi_O = ET_O/TR_O = 1{-}V_J$	Efficiency/probability with which a PSII trapped electron is transferred from $\mathbf{Q_A}$ to $\mathbf{Q_B}$
$\psi_{(RO)} \equiv \psi_O = RE_O/TR_O = 1{-}V_I$	Efficiency/probability with which a PSII trapped electron is transferred until PSI acceptors
$\varphi_{(PO)} = TR_O/ABS \equiv 1{-}F_O/F_M \equiv F_V/F_M$	Maximum quantum yield of primary PSII photochemistry
$\varphi_{(EO)} = ET_O/ABS \equiv [F_V/F_M \times (1{-}V_J)]$	Quantum yield for electron transport the Q_A^- for the electron acceptor intersystem
$\varphi_{(RO)} = RE_O/ABS \equiv (1{-}F_O/F_M) \times (1{-}V_I) \equiv$ $\equiv \varphi_{(PO)} - \psi_{(EO)} - \delta_{(RO)}$	Quantum yield for electron transport (from Q_A^-) to the final electron acceptor of the PSI
$\varphi_{(DO)} = 1{-}\varphi_{(PO)} \equiv F_O/F_M$	Expresses the probability that the energy of an absorbed photon is dissipated as heat
$\delta_{(RO)} = RE_O/\ ET_O \equiv (1{-}V_I)/(1{-}V_J)$	Efficiency/probability with which an electron from $\mathbf{Q_B}$ is transferred until PSI acceptors
$\varrho_{(RO)} = RE_O/TR_O \equiv (1{-}V_I)/(1{-}F_O)$	Efficiency with which an excitation can move an electron within the Q_A^- electron transport chain to the final electron acceptors of the PSI
**Specific energy fluxes (per active PSII reaction center)**	
$ABS/RC = (M_O/V_J) \text{ x } (1 \times \varphi_{(PO)})$	Average absorbed photon flux per PSII reaction center (or also, apparent antenna size of an active PSII)
$TR_O/RC = M_O/V_J$	Maximum trapped exciton flux per PSII
$ET_O/RC = (M_O/V_J) \times (1 - V_J)$	Electron transport flux from Q_A to Q_B per PSII
$RE_O/RC =(M_O/V_J) \times (1 - V_I)$	Electron transport flux until PSI acceptors per PSII
$DI_O/RC = ABS/RC - TR_O/RC$	Dissipated energy flux per RC
**Phenomenological energy fluxes [per excited cross-section (CS) in *t*=0 or *t*=max]**	
RC/CS_O	Relative number of active PSII reaction center per excited cross-section at *t=0*
ABS/CS_O	Absorbed photon flux per cross-section (or also, apparent PSII antenna size) at *t=0*
TR_O/CS_O	Excitation energy flux trapped by PSII of photosynthesizing sample at *t=0*
ET_O/CS_O	Electron flux transported by PSII of a photosynthesizing sample at *t=0*
DI_O/CS_O	Heat dissipation of excitation energy by PSII of photosynthesizing sample CS at *t=0*

RC/CS_M	Relative number of active PSII reaction centers per excited cross-section *t=max*
ABS/CS_M	Absorption flux per cross-section ~F_M at *t=max*
TR_O/CS_M	Maximum trapped exciton flux per cross-section at *t=max*
ET_O/CS_M	Electron transport flux from Q_A to Q_B per cross section at *t=max*
DI_O/CS_M	Heat dissipation of excitation energy by PSII of a photosynthesizing sample cross-section *t=max*
**De-excitation rate constants of PSII antenna**	
$\mathbf{k_N} = \mathbf{k_F} \times (M_O/V_J)/F_M$	Nonphotochemical de-excitation rate constant; $\mathbf{k_F}$ being the rate constant for fluorescence emission
$\mathbf{k_P} = \mathbf{k_N} \times F_V/F_O$	Photochemical de-excitation rate constant
**"Performance" indexes (combination of parameters)**	
$SFI_{(Abs)}$	Structure function index
$PI_{(Abs)}$	Performance index for energy conservation from photons absorbed by PSII antenna, until the reduction of PSI acceptors
$PI_{(CSo)}$	Performance index on cross section basis at *t=0*
$PI_{(CSm)}$	Performance index on cross section basis at *t=max*
**Driving forces of photosynthesis (total driving forces for photochemical activity)**	
$D.F._{(Abs)} = \log (PI_{(Abs)})$	Driving force on absorption basis
$D.F._{(CSo)} = \log (PI_{(CSo)})$	Driving force on cross-section basis initial at *t=0*
$D.F._{(CSm)} = \log (PI_{(CSm)})$	Driving force on cross-section basis final at *t=max*

Subscript "0" indicates that the parameter refers to the onset of illumination—[29]. Strasser, R.J.; Srivastava, A.; Tsimilli-Michael, M. The Fluorescence Transient as a Tool to Characterize and Screen Photosynthetic Samples. In Probing Photosynthesis: Mechanisms, Regulation and Adaptation;1st Eds; CRC Press: London, UK, 2000; pp. 443–480.

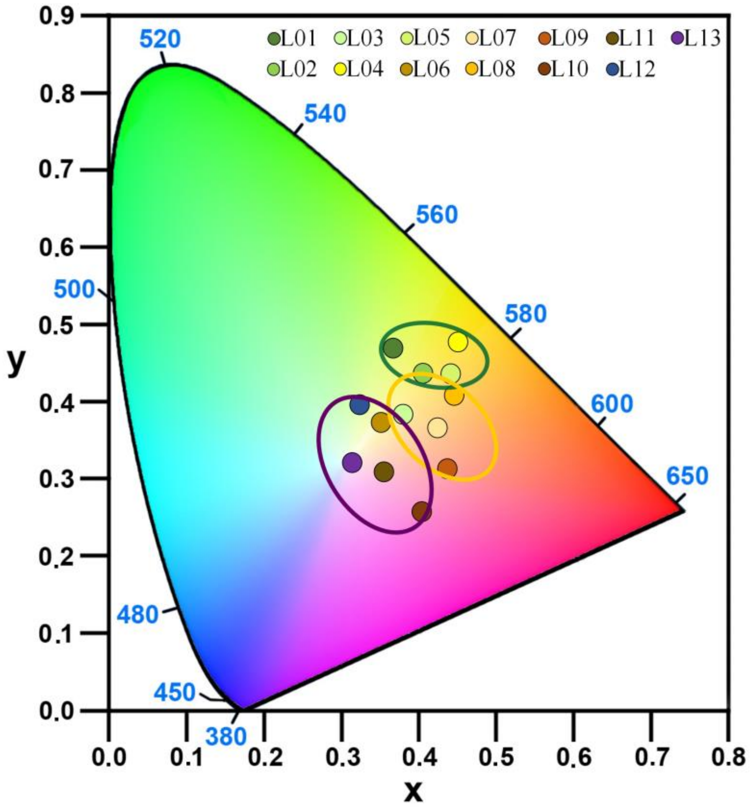

Figure S1. Chromaticity index obtained using single linkage Euclidean distances and the formation of three clusters associated with variegated leaves that are green, yellow, and red-purple.

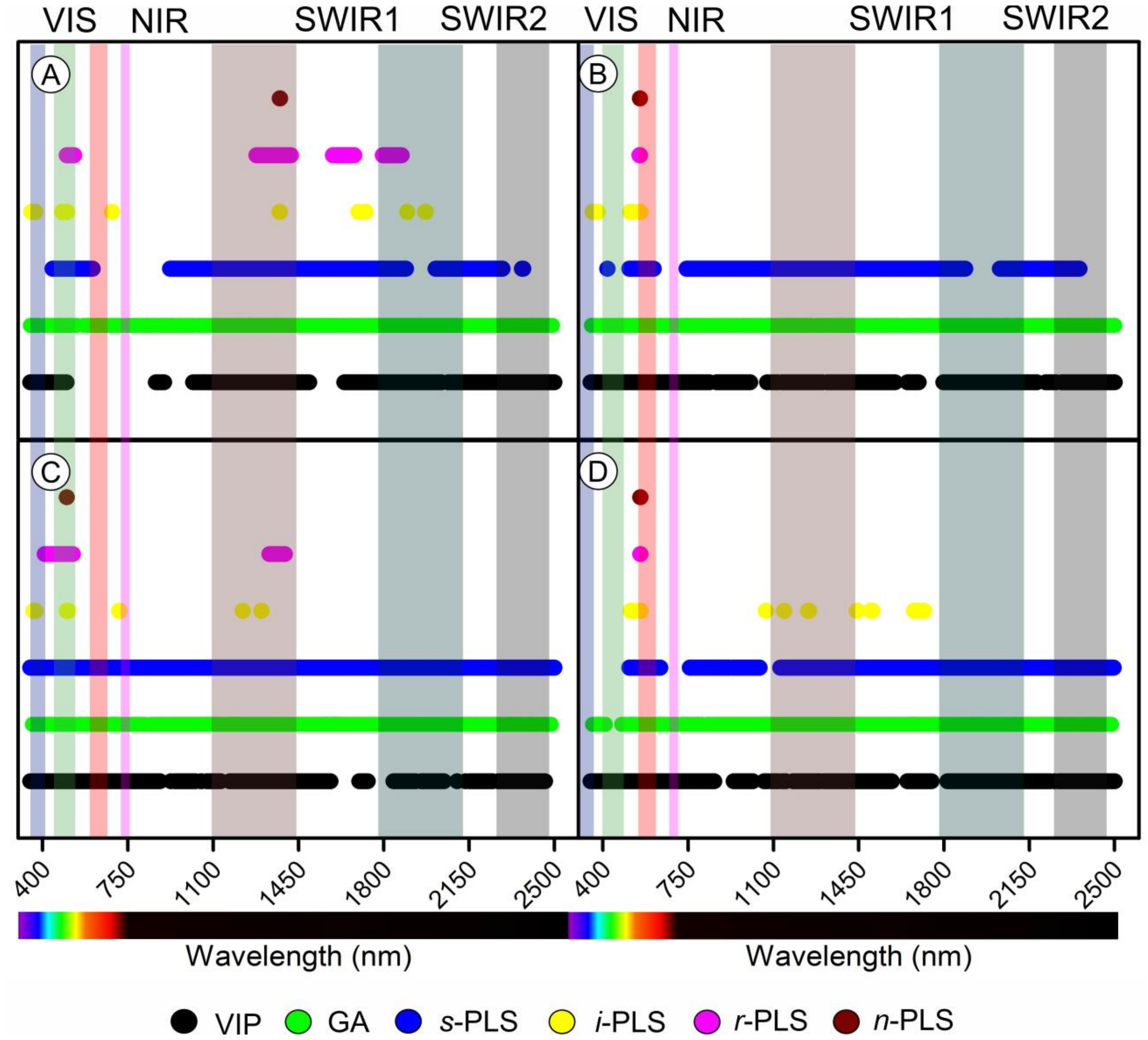

Figure S2. Selected most responsive variables among the wavelengths of 350-2500 nm by VIP, GA, *s*-PLS, *i*-PLS, *r*-PLS, *n*-PLS algorithms for variegated leaves. (**A**) Weight (g). (**B**) Leaf area (m^2). (**C**) Specific leaf area ($cm^2\ g^{-1}$). (**D**) Estimated leaf thickness (μm).

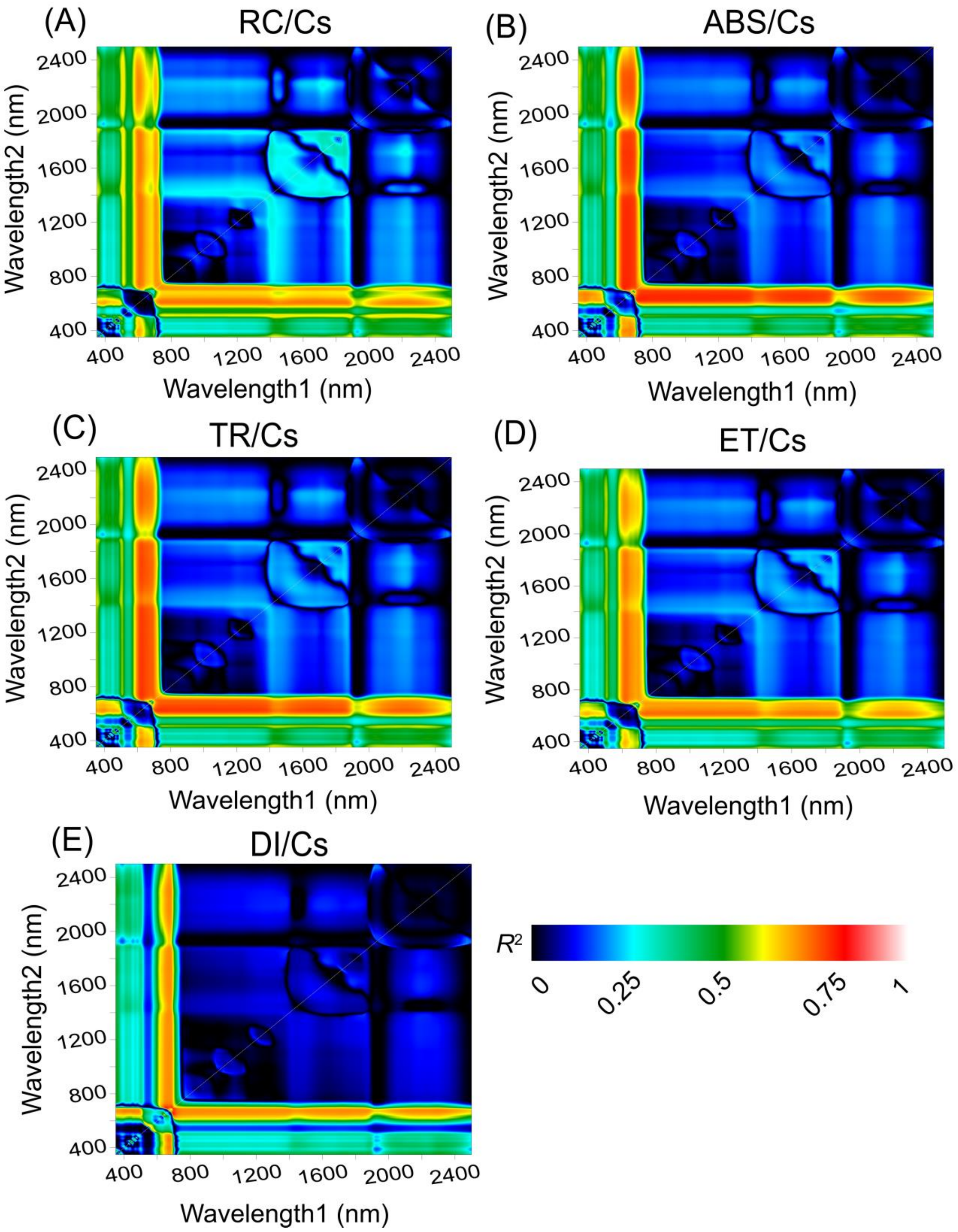

Figure S3. Count plot map of coefficient of correlation (R^2) from the linear regression between phenomenological energy flow through excited cross-sections (CSs) of *Codiaeum variegatum* (L.) A. Juss leaves and wavelengths1 vs wavelength2 for 350 to 2500 nm. (**A**) RC/CS, indicate the % of active/inactive reaction centers. (**B**) ABS/CS, absorption flow by approximate CS; (**C**) TR/CS, energy flow trapped by CS. (**D**) ET/CS, electron transport flow by CS. (**E**) DI/CS, energy flow dissipated by CS. Dark blue to red displayed increased associations.

References

23. Gitelson, A. Nondestructive Estimation of Foliar Pigments (Chlorophylls, Carotenoids, and Anthocyanins) Contents: Evaluating a Semianalytical Three-Band Model. In *Hyperspectral remote sensing of vegetation*; Thenkabail, P.S., Lyon, J.G., Huete, A., Eds.; CRC Press: New York, NY, USA 2011; p. 782.
29. Strasser, R.J.; Srivastava, A.; Tsimilli-Michael, M. The Fluorescence Transient as a Tool to Characterize and Screen Photosynthetic Samples. In *Probing Photosynthesis: Mechanisms, Regulation and Adaptation*;1st Eds; CRC Press: London, UK, 2000; pp. 443–480.
66. Gitelson, A.; Merzlyak, M.N. Spectral Reflectance Changes Associated with Autumn Senescence of *Aesculus hippocastanum* L. and *Acer platanoides* L. Leaves. Spectral Features and Relation to Chlorophyll Estimation. *J. Plant Physiol.* **1994**, *143*, 286–292.
67. Chappelle, E.W.; Kim, M.S.; McMurtrey, J.E. Ratio Analysis of Reflectance Spectra (RARS): An Algorithm for the Remote Estimation of the Concentrations of Chlorophyll A, Chlorophyll B, and Carotenoids in Soybean Leaves. *Remote Sens. Environ.* **1992**, *39*, 239–247.
68. Pontius, J.; Martin, M.; Plourde, L.; Hallett, R. Ash Decline Assessment in Emerald Ash Borer-Infested Regions: A Test of Tree-Level, Hyperspectral Technologies. *Remote Sens. Environ.* **2008**, *112*, 2665–2676.
69. Metternicht, G. Vegetation Indices Derived from High-Resolution Airborne Videography for Precision Crop Management. Int. *J. Remote Sens.* **2003**, *24*, 2855–2877.
70. Blackburn, G.A. Spectral Indices for Estimating Photosynthetic Pigment Concentrations: A Test Using Senescent Tree Leaves. *Int. J. Remote Sens.* **1998**, *19*, 657–675.
71. Merzlyak, M.N.; Chivkunova, O.B.; Solovchenko, A.E.; Naqvi, K.R. Light Absorption by Anthocyanins in Juvenile, Stressed, and Senescing Leaves. *J. Exp. Bot.* **2008**, *59*, 3903–3911.
72. Garbulsky, M.F.; Peñuelas, J.; Gamon, J.; Inoue, Y.; Filella, I. The Photochemical Reflectance Index (PRI) and the Remote Sensing of Leaf, Canopy and Ecosystem Radiation Use Efficiencies. A Review and Meta-Analysis. *Remote Sens. Environ.* **2011**, *115*, 281–297.
73. Lang, M.; Stober, F.; Lichtenthaler, H.K. Fluorescence Emission Spectra of Plant Leaves and Plant Constituents. *Radiat. Environ. Biophys.* **1991**, *30*, 333–347.
74. Stimson, H.C.; Breshears, D.D.; Ustin, S.L.; Kefauver, S.C. Spectral Sensing of Foliar Water Conditions in Two Co-Occurring Conifer Species: *Pinus edulis* and *Juniperus monosperma*. Remote Sens. Environ. **2005**, *96*, 108–118.
75. Apan, A.; Held, A.; Phinn, S.; Markley, J. Formulation and Assessment of Narrow-Band Vegetation Indices from EO-1 Hyperion Imagery for Discriminating Sugarcane Disease. In Proceedings of the Spatial Sciences Institute Biennial Conference (SSC 2003): Spatial Knowledge Without Boundaries, Canberra, Australia 22–27 Sep 2003; pp. 1–13.
76. Nagler, P.L.; Inoue, Y.; Glenn, E.P.; Russ, A.L.; Daughtry, C.S.T. Cellulose Absorption Index (CAI) to Quantify Mixed Soil–Plant Litter Scenes. Remote Sens. Environ. **2003**, *87*, 310–325.
77. Serrano, L.; Peñuelas, J.; Ustin, S.L. Remote Sensing of Nitrogen and Lignin in Mediterranean Vegetation from AVIRIS Data: Decomposing Biochemical from Structural Signals. Remote Sens. Environ. **2002**, *81*, 355–364.

Disclaimer/Publisher's Note: The statements, opinions and data contained in all publications are solely those of the individual author(s) and contributor(s) and not of MDPI and/or the editor(s). MDPI and/or the editor(s) disclaim responsibility for any injury to people or property resulting from any ideas, methods, instructions or products referred to in the content.
